# Background and clinical significance of biomarker-based patient enrichment in non-small-cell lung cancer drug development

**DOI:** 10.1038/s41598-024-57556-3

**Published:** 2024-03-26

**Authors:** Kenji Harada, Shunsuke Ono

**Affiliations:** 1 Laboratory of Pharmaceutical Regulatory Science, Graduate School of Pharmaceutical Sciences, The University of Tokyo, 7-3-1 Hongo, Bunkyo-ku, Tokyo, 113-0033 Japan; 2 Kyowa Kirin Co., Ltd., Tokyo, Japan

**Keywords:** Biomarkers, Drug development, Non-small-cell lung cancer, Drug discovery, Biomarkers, Business strategy in drug development, Non-small-cell lung cancer, Clinical trials

## Abstract

Pharmaceutical companies have adopted biomarker-based enrichment (personalized) strategies to improve research and development productivity. We explored the background in which personalized strategies are adopted and examined whether their adoption is linked to improved efficacy of new drugs approved for non-small cell lung cancer (NSCLC) by US Food and Drug Administration (FDA). We extracted data from the first labels of drugs approved for NSCLC between May 2003 and February 2021, and performed a qualitative comparative analysis and meta-analysis. Personalized strategies were adopted in more than half of the trials (16/27) and were often used in trials aimed at obtaining first-line indications and in drugs that were not first-in-class. The meta-analysis showed that personalized trials had significantly improved progression-free survival (PFS) hazard ratio (HR) than trials without personalization but not for relative response rate ratio (RRR) or overall survival (OS) HR. Trials in which PFS HR was the primary endpoint tended to have improved PFS HR, and trials in which OS HR was the primary endpoint had worse PFS HR. The efficacy endpoints that are substantially affected by personalized strategies appear to differ, especially for new drugs with novel mechanism of action (MOA), because trial designs are employed to validate drug-specific advantages.

## Introduction

The recent rise in pharmaceutical Research & Development (R&D) costs and the decline in the number of approved drugs have long called for the need to improve drug development efficiency^1,2^. Approaches based on pharmacogenomics (PGx), a field of research that clarifies the causal relationship between drug response and genetic polymorphisms in organisms, are expected to optimize the efficacy and safety profile of drugs based on individual genome information^3^. The practical application of PGx is a personalized approach that uses biomarkers in clinical trials. There are several drugs for which the patient enrichment biomarkers specified by PGx are listed on the drug label^4^. Examples include crizotinib and dabrafenib. Crizotinib is a kinase inhibitor indicated for the treatment of adult patients with metastatic NSCLC whose tumors are anaplastic lymphoma kinase (ALK) or ROS1-positive as detected by an FDA-approved test. Dabrafenib is a kinase inhibitor indicated for the treatment of patients with unresectable or metastatic melanoma with BRAF V600E mutation as detected by an FDA-approved test.

The recent emergence of anticancer drugs with innovative mechanisms of action has significantly changed the methods and strategies for new drug development. For example, immune checkpoint inhibitors, such as PD-1/PD-L1 inhibitors, have dramatically improved the prognosis of patients with cancer^5^. The mode of cancer treatment is also changing, as evidenced by extensive advances in gene therapy. In this increasingly competitive anticancer drug market, it is natural for companies to conduct clinical development to demonstrate the most obvious advantages of their products based on their characteristics. The application of personalized strategies to new drug development serves as the objective of companies.

A previous study analyzing FDA-approved anticancer drugs found that the adoption of personalized strategies is associated with improved efficacy^6^. In addition, studies on the impact of personalized strategies on drug development showed that the phase transition rates were higher for products using patient enrichment biomarkers than for those not using them^7^ and that personalized strategies were associated with higher median response rates (RR) and longer median PFS in phase I and II trials^8,9^.

However, from the perspective of optimizing the pharmaceutical business, companies do not unconditionally apply personalization strategies to all drugs. Personalized strategies necessarily involve some type of market segmentation that may reduce potential future revenue. Strategies that seek competitive advantages of a drug other than its high efficacy (e.g., improved safety) may also be reasonable in certain therapeutic areas. Pharmaceutical companies are expected to use personalized strategies not only to improve the efficacy observed in clinical trials, but also to optimize the positioning of their products in individual markets.

In this study, we investigated the adoption of personalized strategies in recent pivotal and/or phase III trials of anticancer drugs approved in the US for the treatment of NSCLC, one of the cancers to which personalized strategies are most applied. Since NSCLC has high genetic heterogeneity and the prognosis and safety of treatment with existing drugs are still insufficient, the application of personalized medicine (i.e., patient selection by biomarker) has been pursued. Furthermore, it has become clear that drugs with PD-1/L1 inhibition as described above are effective for NSCLC. We applied logical analysis and identified the conditions in which companies adopted personalized strategies. We then examined the relationship between personalized strategies and the effect size in these trials. As several efficacy endpoints have been examined in oncological trials, we explored whether the observed relationship was the same across different endpoints. Based on the results of these analyses, we discuss what should be considered when interpreting and generalizing the results of pivotal trials conducted to obtain regulatory approval.

## Methods

**Figure 1:**
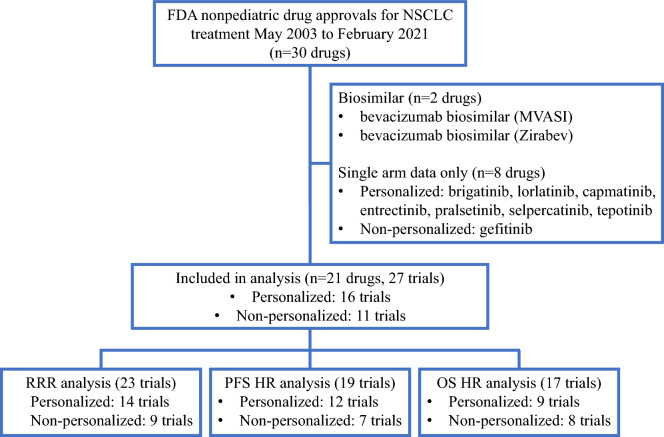
Flow diagram of search strategy and study selection.

All anti-cancer drugs approved for NSCLC between May 2003 and February 2021 were identified by the FDA^10^. In 2003, gefitinib was approved as the first molecularly targeted drug for the treatment of NSCLC. Among these drugs, those listed in the FDA’s Table of Pharmacogenomic Biomarkers in Drug Labeling as of February 2021, and those with a patient enrichment biomarker listed in the “Indication and usage” section of the label were classified as anticancer drugs, for which a personalized strategy was adopted. We reviewed the first label approved for the treatment of NSCLC and extracted data on efficacy endpoints, including RR, PFS, and OS. There were six drugs for which results with and without a personalized strategy were indicated. For drugs that received accelerated approval, we used data at the time of final approval. Drugs with only single-arm trials listed on the label were excluded from the analysis because it is difficult to reliably estimate the effect size (e.g., risk ratio, HR) (Fig. 1). Biosimilars were also excluded from the analysis because they basically follow the personalized/non-personalized strategy of their predecessors and could not choose the personalized/non-personalized strategy itself as a strategic option in drug development (Fig. 1). Informed consent was not sought for this study because it is not a research involving human participants.

To classify the background of a company’s choice to adopt a personalized/non-personalized drug strategy, we performed qualitative comparative analysis (QCA). QCA uses set theory and Boolean algebra to infer the causal relationship between the characteristics of individual cases and their results, enabling a logical examination of similarities and differences among cases. The QCA results are expressed in the form of multiple conditional expressions that provide clues for deciphering causal relationships. After reviewing the “Indication and usage” section of the labels, we obtained the following six background conditions for each drug that may have contributed to the decision to adopt a personalized strategy:(1) whether the personalized strategy has been taken in other cancers before development in NSCLC, (2) whether the drug has indication for squamous NSCLC only (this cancer type is known to have few genetic mutations), (3) whether the drug has received orphan designation, (4) whether the drug is an immune checkpoint inhibitor (i.e., a new MOA), (5) whether the drug is the first anticancer drug targeting the molecule in NSCLC, (6) whether the drug has a first-line indication. The logical remainder (i.e., the parsimonious solution) was applied to the QCA. Tosmana ver. 1.6.1.0) was used for statistical analysis.

We performed a meta-analysis (random-effects model) to determine the association between personalized strategies and effect size. The effect size of the HR for PFS and OS and the relative response rate ratio (RRR) for RR were calculated. The mediators or confounders included personalization, randomization, drug type (cytotoxic vs. targeted), concomitant medications, control drug (active treatment vs. placebo), crossover, tumor immunity-related target, FDA approval date (before the median vs. after), effect size pertaining to the primary endpoint of the trial, first-line treatment, first-in-class target, priority review, and orphan drug designation. Independent samples were assessed using the Wilcoxon rank-sum test.

We further investigated the effect sizes of PFS and OS using meta-regression analysis (random-effects model) with independent variables (for PFS: personalization, OS HR pertains to the primary endpoint; for OS: drug type, tumor immunity-related target, and priority review), all of which were significantly associated with PFS HR and OS HR in each meta-analysis.

Differences were considered statistically significant at *p* < 0.05. Statistical analyses were performed using Stata 14 software (StataCorp, College Station, TX, USA).

## Results

We identified 21 drugs approved for the treatment of NSCLC between May 2003 and February 2021 that met the criteria described in the Methods section (Fig. 1). For the 21 drugs, 27 trials were phase III and/or pivotal trials, 16 were personalized trials, and 11 were non-personalized trials (Supplemental Tables 1 and 2). All drugs, except for paclitaxel protein-bound particles and pemetrexed disodium, were molecularly targeted drugs, the targets of which were epidermal growth factor receptor (EGFR)-related targets (EGFR, human epidermal growth factor (HER)2, and HER4), anaplastic lymphoma kinase (ALK), immuno-onco-targets (Programmed death receptor-1/Programmed cell Death ligand 1 and Cytotoxic T-lymphocyte associated antigen-4), and others (vascular endothelial growth factor-2, v-raf murine sarcoma viral oncogene homolog B1, and mitogen-activated extracellular signal-regulated kinase 1/2).

**Table 1:** Comparison of the main characteristics between personalized and nonpersonalized trials * *P* < 0.1, ** *P* < 0.05, *** *P* < 0.01 ♱ Wilcoxon rank-sum test (two-sided). ‡ Cutoff used was the median of distribution.

	All (*n* = 27)			RRR (*n* = 23)			PFS (*n* = 19)			OS (*n* = 17)		
Characteristic	Personalized trials (*n* = 16)	Non-personalized trials (*n* = 11)	*p* value ♱	Personalized trials (*n* = 14)	Non-personalized trials (*n* = 9)	*p* value ♱	Personalized trials (*n* = 12)	Non-personalized trials (*n* = 7)	*p* value ♱	Personalized trials (*n* = 9)	Non-personalized trials (*n* = 8)	*p* value ♱
Trial design, No												
Randomized	14	11	0.232	12	9	0.246	12	7	NA	9	8	NA
Nonrandomized	2	0		2	0		0	0		0	0	
Class agent, No												
Targeted	16	9	0.082*	14	7	0.071*	12	6	0.190	9	7	0.289
Cytotoxic	0	2		0	2		0	1		0	1	
﻿Treatment, No												
Combination	6	5	0.685	4	4	0.446	2	3	0.224	4	3	0.778
Single agent	10	6		10	5		10	4		5	5	
Control arm, No												
Active treatment	16	9	0.082*	14	7	0.071*	12	5	0.057*	9	7	0.289
Placebo/BSC	0	2		0	2		0	2		0	1	
Crossover allowed, No												
Yes	3	2	0.971	3	2	0.965	3	2	0.868	2	1	0.611
No	13	9		11	7		9	5		7	7	
﻿Target, No												
Immuno-onco target	5	4	0.786	3	3	0.535	3	2	0.868	5	2	0.215
Non-Immuno-onco target	11	7		11	6		9	5		4	6	
Approval date‡												
2016/10/24 ~ 2021/2/22	12	2	0.004***	10	2	0.024**	8	2	0.118	6	0	0.005***
2004/8/19 ~ 2016/10/23	4	9		4	7		4	5		3	8	
Primary endpoint, No												
Include RRR	3	2	0.971	3	2	0.965	1	1	0.691	0	0	NA
Not include RRR	13	9		11	7		11	6		9	8	
Primary endpoint, No												
Include PFS HR	12	2	0.004***	12	2	0.003***	12	2	0.001***	7	1	0.009***
Not include PFS HR	4	9		2	7		0	5		2	7	
Primary endpoint, No												
Include OS HR	5	7	0.103	3	5	0.101	3	4	0.173	5	7	0.162
Not include OS HR	11	4		11	4		9	3		4	1	
Therapeutic line, No												
First	14	4	0.007***	12	3	0.012**	10	2	0.020**	8	2	0.010***
Not first	2	7		2	6		2	5		1	6	
First in class for the target, No												
Yes	4	7	0.049**	3	5	0.101	1	3	0.083*	2	6	0.035**
No	12	4		11	4		11	4		7	2	
Priority review, No												
Yes	12	5	0.125	10	4	0.206	8	3	0.324	6	3	0.243
No	4	6		4	5		4	4		3	5	
Orphan Drug Designation, No												
Yes	9	2	0.052*	9	2	0.054*	7	2	0.223	2	2	0.896
No	7	9		5	7		5	5		7	6

**Table 2:** QCA analysis results with parsimonious solution.

Personalization	Formula No	Explanatory formula	Drug
Yes	(1)	Indication is squamous NSCLC only {No} and Orphan drug designation {Yes}	Afatinib, alectinib, ceritinib, crizotinib, dabrafenib, dacomitinib, gefitinib, osimertinib, trametinib
	(2)	The personalized strategy is used prior to NSCLC indication in the US {No} and Immuno-oncology target {Yes} and First line {Yes}	atezolizumab, cemiplimab, ipilimumab, nivolumab
	(3)	Indication is squamous NSCLC only {No} and Immuno-oncology target {No} and First in class for the target {No}	Afatinib, alectinib, ceritinib, dacomitinib, erlotinib, gefitinib, osimertinib, ramucirumab
	(4)	Orphan drug designation {No} and Immuno-oncology target {No} and First in class for the target {No}	erlotinib, ramucirumab
	(5)	Orphan drug designation {Yes} and First in class for the target {Yes}	Crizotinib, dabrafenib, trametinib
No	(6)	Indication is squamous NSCLC only {Yes}	Afatinib, necitumumab, nivolumab
	(7)	The personalized strategy is used prior to NSCLC indication in the US {Yes} and Immuno-oncology target {Yes}	Pembrolizumab
	(8)	Orphan drug designation {No} and First line {No}	Atezolizumab, erlotinib, nivolumab, pemetrexed disodium, ramucirumab
	(9)	Orphan drug designation {No} and Immuno-oncology target {No} and First in class for the target {Yes}	bevacizumab, erlotinib, paclitaxel protein-bound particle, pemetrexed disodium, ramucirumab
	(10)	Immuno-oncology target {Yes} and First line {No}	Atezolizumab, durvalumab, nivolumab
	(11)	First in class for the target {Yes} and First line {No}	Atezolizumab, erlotinib, nivolumab, pemetrexed disodium, ramucirumab

### Descriptive characteristics of personalized and non-personalized trials that met the selection criteria

The characteristics of personalized and non-personalized trials that met the selection criteria are summarized and compared in Table 1. Recently, the number of personalized trials has increased. Personalized trials were performed only for molecularly targeted drugs (100% [16/16]) and mostly for drugs that were not first-in-class (75% [12/16] vs. 36% [4/11]). They were administered more often as first-line drugs (88% [14/16] vs. 36% [4/11]) and orphan drugs (56% [9/16] vs. 18% [2/11]). Placebo controls were not used in personalized trials. Personalized trials more often adopted the PFS HR as the primary endpoint (75% [12/16] vs. 18% [2/11]), whereas non-personalized trials tended to use the OS HR as the primary endpoint (31% [5/16] vs. 64% [7/11]).

These findings were consistent when viewed separately for each endpoint employed in the study (Table 1). PFS was the primary endpoint in all personalized trials in which it was evaluated.

The results of the logical analysis (QCA) of the background of the personalized and non-personalized trials are shown in Table 2. The truth table is presented in Supplementary Table 3. Two trials (personalized pembrolizumab and non-personalized durvalumab) were acknowledged as exceptional cases.

The backgrounds of the trials with and without personalized strategies were classified into five and six categories, respectively ((1)–(5) and (6)–(11) in Table 2). No personalized trials have been conducted for squamous NSCLC. Personalized strategies have been employed in trials for first-line applications and follow-on trials (i.e., not first-in-class) drugs. Orphan designation is generally linked to the conduct of personalized trials. Orphan drug background was associated with the conduct of personalized trials, not alone, but in conjunction with other backgrounds.

### Effect sizes and personalized strategies for each endpoint (meta-analysis)

**Figure 2:**
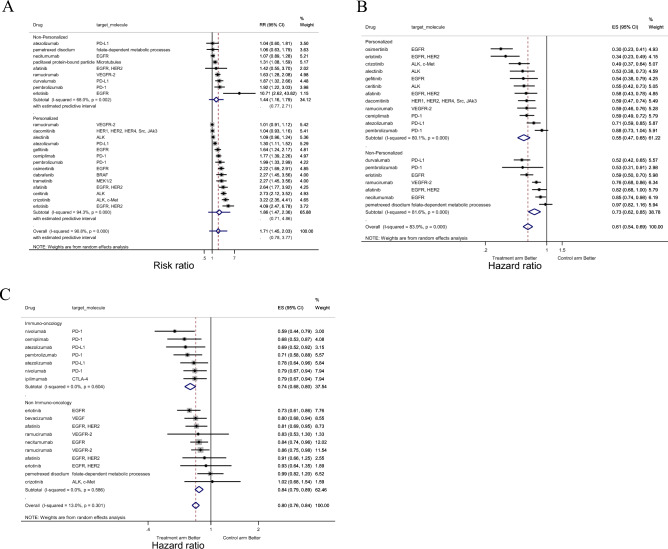
Forest-plot representing the relative response rate ratio (RRR) (**A**), hazard ratios (HRs) for progression-free survival (PFS) (**B**), and HRs for overall survival (OS) (C) between the experimental and control arms by personalized therapy status ((**A**), (**B**)), or by imuno-onco target status (**C**) in trials. In (**A**), RRR is shown and lines to the right of the vertical line indicate improvement in the experimental arm. In (**B** and **C**), the plots show HRs and, therefore, lines to the left of the vertical line indicate improvement (i.e., lower HR for PFS or OS) for the experimental arm.

The results of meta-analysis are presented in Fig. 2 and Table 3.

**Table 3:** Univariate and multivariate meta-analyses according to trial characteristics using a random effects model.

	RRR			PFS HR			OS HR		
	Coefficient	SE	*P* value	HR	SE	*P* value	HR	SE	*P* value
Personalized status: ﻿Non-personalized									
Personalized	0.22	0.20	0.273	0.76	0.10	0.048 **	0.97	0.05	0.532
Personalized (meta-regression) ♱				0.85	0.10	0.174			
Study design: ﻿Nonrandomized									
Randomized	-0.30	0.35	0.39	NA	NA	NA	NA	NA	NA
Class of agent: Cytotoxic									
Targeted	0.39	0.33	0.246	0.61	0.17	0.086*	0.80	0.08	0.042**
﻿Single agent: Yes									
No	-0.24	0.19	0.216	1.21	0.18	0.222	1.04	0.06	0.443
﻿Control arm: Placebo									
Active treatment	-0.49	0.40	0.238	1.11	0.24	0.649	1.11	0.11	0.303
﻿Cross-over allowed: No									
Yes	0.26	0.22	0.256	0.82	0.13	0.204	1.04	0.07	0.594
Target molecule: Non-Immuno-onco target﻿									
Immuno-onco target	-0.08	0.22	0.701	1.09	0.17	0.606	0.88	0.04	0.023**
Approval date‡: Aug 19th, 2004 ~ Oct 23rd, 2016﻿									
Oct 24th, 2016 ~ Feb 22nd, 2021	-0.08	0.19	0.678	0.87	0.12	0.332	0.93	0.05	0.178
Primary endpoint: Not include RRR﻿									
Include RRR	0.06	0.23	0.783	0.87	0.23	0.612	NA	NA	NA
Primary endpoint: Not include PFS HR﻿									
Include PFS HR	0.12	0.20	0.559	0.75	0.11	0.062*	0.98	0.06	0.702
Primary endpoint: Not include OS HR﻿									
Include OS HR	-0.21	0.20	0.292	1.43	0.16	0.004***	0.92	0.07	0.277
Include OS HR (meta-regression) ♱				1.36	0.15	0.016 **			
Therapeutic line: Not first line﻿									
first line	-0.01	0.21	0.955	0.87	0.12	0.314	1.02	0.06	0.727
First in class for the target: No﻿									
Yes	0.10	0.21	0.626	1.17	0.19	0.335	1.01	0.06	0.812
﻿Priority review: No									
Yes	0.13	0.19	0.51	0.86	0.12	0.291	0.87	0.04	0.013**
Orphan Drug Designation: No﻿									
Yes	0.10	0.19	0.625	0.89	0.12	0.405	1.07	0.07	0.268

Baseline category is underlined.﻿ RRR = relative response rate ratio; PFS = progression-free survival; OS = overall survival; HR = hazard ratio * *P* < 0.1, ** *P* < 0.05, *** *P* < 0.01 ♱ Meta-regression analysis was performed including only factors considered statistically significant in univariate analysis. ‡ Cutoff used was the median of distribution.

Adoption of a personalized strategy was associated with improved PFS HR (HR, 0.76; *p* = 0.048) but not with RRR or OS HR (Table 3). For PFS HR, being a molecular-targeted drug (HR, 0.61; *p* = 0.086) and having a primary endpoint of PFS HR (HR, 0.75; *p* = 0.062) also tended to be associated with an improved PFS HR. In contrast, having a primary endpoint of OS HR was associated with worse PFS HR (HR, 1.43; *p* = 0.004). Being a molecular-targeted drug (HR, 0.80; *p* = 0.042), being an immune checkpoint inhibitor (HR, 0.88; *p* = 0.023), and priority review (HR, 0.87; *p* = 0.013) were associated with improved OS HR. Heterogeneity between the trials for RRR and PFS HR was classified as high (I^2^ > 75%) or low (I^2^ < 50%) for OS HR.

Meta-regression analysis with two independent variables showing a significant association in the meta-analysis of PFS HR indicated that trials with OS HR as the primary endpoint were associated with worse PFS HR (HR, 1.36; *p* = 0.016).

## Discussion

We analyzed clinical trials that have been submitted as the basis for regulatory approval for NSCLC since 2003 to evaluate the context in which personalized strategies have been adopted and whether these strategies improve the efficacy observed in such trials.

Personalized strategies were adopted in more than half of the trials (16/27 [59%]). Personalized trials were more likely to include the HR for PFS as the primary endpoint, whereas non-personalized trials included the HR for OS as the primary endpoint. The analysis also revealed that personalized strategies are often used in trials aimed at obtaining first-line indications and in trials of drugs that are not first-in-class. QCA also revealed that overall, the orphan drug designation status is associated with the adoption of personalized strategies and may also be a contributing factor to the relationship between other background factors and the adoption of personalized strategies.

Our meta-analysis showed a significant positive correlation between personalized strategies and improvement in PFS HR, but not in RRR or OS HR. Our results also revealed that the relationship between personalized strategies and outcomes varies depending on what is employed as the primary endpoint of the trial: in trials where PFS was the primary endpoint, PFS HR improved with the adoption of personalized strategies, whereas in trials where OS was the primary endpoint, the improvement did not occur. New drugs with tumor immune-related targets that have not been investigated in previous studies have shown a clear improvement in OS, indicating that the emergence of such drugs may weaken the correlation between personalized strategies and effect sizes, particularly for OS HR.

The QCA results, which enable logical linking of the background conditions of trials to the adoption of personalized designs, provide interesting clues for inferring why a company would (or would not) conduct personalized trials. These results could also help explain the consistent distribution patterns shown in Table 1.

Several combinations of conditions were found to correspond to the use or non-use of personalized strategies, and these combinations led to the following interpretation: First, a personalized strategy was adopted when the target indication was NSCLC with targeted driver oncogene mutations^11–14^ ((1) and (6) in Table 2). Second, personalized strategies tend to be adopted in trials aimed at obtaining approval for first-line treatments ((2), (8), (10), and (11) in Table 2). This finding may reflect the fact that patients eligible for first-line treatment are more heterogeneous than those eligible for second-line or subsequent treatment and that the magnitude of the efficacy signal expected for approval is greater for first-line treatment than for second-line treatment. Third, personalized strategies are often used in trials for drugs that are not first-in-class (i.e., follow-on drugs (3), (4), (9), and (11) in Table 2). In the development of first-in-class drugs, companies tend to approach broader patient populations, but there may be a lack of validated and appropriate biomarkers. In contrast, companies developing follow-on new drugs with the same mechanism of action can take advantage of richer, accumulated information on patient selection. The existence of treatment-resistant patients is gradually becoming more evident, leading to a growing need for personalization through the appropriate use of biomarkers. In some cases, if the preceding drug has adopted a personalized strategy, follow-on products may have no choice but to adopt the same strategy^15,16^. Fourth, personalized strategies are more likely to be adopted when the target indication is given orphan status ((1), (5), (8), and (9) in Table 2). This relationship naturally arises because orphan designation is usually provided when attempts are made to subdivide patients with NSCLC using biomarkers.

Our observation that the background of personalization through the use of biomarkers can be classified into several patterns reflects the reality of the recent development strategies of industries, regulatory approval policies, and trends relevant to anticancer drugs. The results can also be seen as an example of the successful exploitation of the potential of recent drugs with distinctive characteristics and a delicate balance of risk–benefit profiles.

Regardless of the background and purpose of personalization, we expect it to improve efficacy and/or safety in clinical trials. A previous study^6^ reported that the adoption of a personalized strategy was significantly correlated with improvements in RRR and HR for PFS and that a positive correlation with improvements in HR for OS was also observed, but weakly (*p* = 0.07). Our results showed that the personalized strategy was associated only with the HR for PFS, and not with the RRR or HR for OS. The observed differences can be explained by the different types of drugs and clinical trials analyzed.

Our results may reflect the inclusion of newly introduced drugs with distinctive features in terms of their mechanisms of action. Compared to previous studies, the targeted drugs in this study included a larger number of drugs that, due to resistance to treatment, may be evaluated more favorably by PFS than by OS. Several studies have suggested that drugs that exert effects on specific molecules in the body may be highly effective in the early stages of treatment and improve PFS HR; however, with continued treatment, drug resistance may develop due to the acquisition of resistance mutations, and OS HR may not improve^17–21^. The samples in the previous study^6^ were anticancer drugs approved between September 1998 and June 2013 and did not include second-generation EGFR tyrosine kinase inhibitors (TKIs) (afatinib), third-generation EGFR TKIs (osimertinib), or each generation of ALK inhibitors (crizotinib, ceritinib, and alectinib), which are known to acquire resistance in clinical settings^22,23^.

Immune checkpoint inhibitors have not been fully analyzed in previous studies^6^. These inhibitors cause the acquisition of tumor immunity by immune cells in patients and the long-term retention of antitumor activity^24–26^. They showed improved OS HR in this study, and this advantage may have masked potential efficacy gains from personalized strategies. In fact, 44.4% (4/9) of trials of immune checkpoint inhibitors in our sample achieved efficacy levels sufficient for approval without a personalized strategy.

The finding that the relationship between personalized strategy and effect size varied by endpoint may also be explained by the fact that we examined only trials that provided evidence to justify successful marketing approval. Our analysis showed that the PFS HR was improved in trials in which PFS was the primary endpoint and worse in trials in which OS was the primary endpoint (Table 3). This indicates that the observed efficacy for each endpoint was used flexibly as a basis for approval, depending on the type of drug and its indication.

The endpoints emphasized in regulatory approval have also changed over time. Approval for NSCLC used to be granted based on trials with OS as the primary endpoint. However, the discussion at the Oncologic Drugs Advisory Committee (ODAC) on December 3, 2003, concluded that PFS may be used as an endpoint to evaluate drug effects in metastatic disease for consideration of regular approval. The FDA issued guidance^27^ based on the discussion at the ODAC to encourage the use of PFS as the primary endpoint and as the basis for approval. In contrast, recent evaluations of immune checkpoint inhibitors have focused more on OS than on PFS as an endpoint owing to their unique mechanism of action. They may cause pseudo progression^5,28^, which results in transient disease progression and worsening of PFS HR even when the OS HR eventually improves.

As seen from the background analysis of the trials mentioned above, a personalized strategy appears to be adopted as an option for a company’s development and life cycle management. Six drugs in our sample had both personalized and non-personalized indications. First, afatinib and pembrolizumab obtained approval for personalized indications and then for non-personalized indications. In contrast, atezolizumab, erlotinib, nivolumab, and ramucirumab were approved for non-personalized indications first and then obtained approval for personalized indications. The effect sizes of the latter indications for each drug were not necessarily large (Supplementary Table 2).

Although it is unclear how much of the relationship found in the study can be attributed to the pharmacological response of drugs related to indications and to the background of trials (i.e., successful trials used to obtain marketing approval), it has been revealed that background factors in the choice of study design may confound the results of efficacy. When comparing drug effect sizes or extrapolating trial results, one must be cautious about the purpose and context of the trial.

The results of this study seem to support the appropriate adoption of a personalized strategy to obtain a favorable effect size and present compelling evidence for regulators. This study does not necessarily provide an answer to the question of whether selecting a personalized approach is appropriate as an industrial development strategy. Nevertheless, since 2003, more than half of the molecular-targeted drugs approved for NSCLC have used personalized strategies for different purposes, suggesting that companies can benefit from strategic options in their drug development. In a competitive environment, biomarker-based personalization will not merely create a downside to market fragmentation, but will also ensure the proper positioning of drugs in the anticancer treatment and pharmaceutical business.

Therefore, it is critical to discover and develop superior biomarkers that enable more efficient and effective enrichment and utilization in cancer treatment. Especially in immuno-oncology, not only the expression of target antigens, but also the tumor mutation burden and the degree of infiltration of immune cells (CD8 + T cells) into the tumor environment may be useful biomarkers^29,30^.

## Conclusions

The current analysis of clinical trials that formed the basis for the approval of NSCLC indications revealed that whether a personalized strategy (biomarker-based enrichment) is adopted in trials is associated with background factors, such as the drug’s MOA and competition from similar drugs in the market. The impact of adopting personalized strategies on endpoint outcomes depends on the characteristics of the drug and its indication, especially for recently developed drugs with novel MOA profiles, as trial designs are often employed to clearly validate such drug-specific advantages. When assessing the relationship between personalized strategies and efficacy, careful consideration should be given to the context in which the clinical trials were conducted.

### Supplementary Information

Supplementary Tables.

## Data Availability

Data available from the corresponding author (shun-ono@mol.f.u-tokyo.ac.jp) upon request.
