# Supplementary material for: Background and clinical significance of biomarker-based patient enrichment in non-small-cell lung cancer drug development
Source: Sci Rep. 2024 Mar 26;14:7194. doi: 10.1038/s41598-024-57556-3 (PMC10966045; doi:10.1038/s41598-024-57556-3)
Supplement: Supplementary file 1 — Supplementary Tables. [file 41598_2024_57556_MOESM1_ESM.docx]

| No. | Drug | FDA approval date | Target molecule | Biomarker as eligibility criteria | Allocation | Class of agent | Concomitant medications | Control drug | Cross over |
| --- | --- | --- | --- | --- | --- | --- | --- | --- | --- |
| 1 | afatinib | 2013/7/12 | EGFR, HER2 | EGFR (exon19 deletions or exon21(L858R) mutations) | Randomized | Targeted | no | Active treatment | no |
| 2 | afatinib | 2016/4/15 | EGFR, HER2 | no | Randomized | Targeted | no | Active treatment | no |
| 3 | alectinib | 2017/11/6 | ALK | ALK | Randomized | Targeted | no | Active treatment | no |
| 4 | atezolizumab | 2016/10/18 | PD-L1 | no | Randomized | Targeted | no | Active treatment | no |
| 5 | atezolizumab | 2018/12/6 | PD-L1 | no EGFR or ALK genomic aberrations | Randomized | Targeted | yes | Active treatment | no |
| 6 | bevacizumab | 2006/10/11 | VEGF | no | Randomized | Targeted | yes | Active treatment | no |
| 7 | cemiplimab | 2021/2/22 | PD-1 | high PD-L1 expression [TPS≥50%]  no EGFR, ALK or ROS1 abrrations | Randomized | Targeted | no | Active treatment | yes |
| 8 | ceritinib | 2017/5/26 | ALK | ALK | Randomized | Targeted | no | Active treatment | no |
| 9 | crizotinib | 2013/11/20 | ALK, c-Met | ALK | Randomized | Targeted | no | Active treatment | yes |
| 10 | dabrafenib | 2017/6/22 | BRAF | BRAF V600E mutation | non-randomized | Targeted | yes | Active treatment | no |

Supplementary Table 1. List and the main characteristics of included FDA Drug Approvals

| No. | Drug | FDA approval date | Target molecule | Biomarker as eligibility criteria | Allocation | Class of agent | Concomitant medications | Control drug | Cross over |
| --- | --- | --- | --- | --- | --- | --- | --- | --- | --- |
| 11 | dacomitinib | 2018/9/27 | HER1, HER2, HER4, Src, JAk3 | EGFR (exon19 deletions or exon21(L858R) mutations) | Randomized | Targeted | no | Active treatment | no |
| 12 | durvalumab | 2018/2/16 | PD-L1 | No | Randomized | Targeted | no | Placebo/BSC | no |
| 13 | erlotinib | 2004/11/18 | EGFR | No | Randomized | Targeted | no | Placebo/BSC | no |
| 14 | erlotinib | 2013/5/14 | EGFR | EGFR (exon19 deletions or exon21(L858R) mutations) | Randomized | Targeted | no | Active treatment | no |
| 15 | gefitinib | 2015/7/13 | EGFR | EGFR (exon19 deletions or exon21(L858R) mutations) | Randomized | Targeted | no | Active treatment | no |
| 16 | ipilimumab | 2020/5/15 | CTLA-4 | expressing PD-L1 [≥1%]  no EGFR or ALK genomic aberrations | Randomized | Targeted | yes | Active treatment | no |
| 17 | necitumumab | 2015/11/24 | EGFR | No | Randomized | Targeted | yes | Active treatment | no |
| 18 | nivolumab | 2015/3/4 | PD-1 | No | Randomized | Targeted | no | Active treatment | no |
| 19 | nivolumab | 2020/5/15 | PD-1 | expressing PD-L1 [≥1%]  no EGFR or ALK genomic aberrations | Randomized | Targeted | yes | Active treatment | no |
| 20 | osimertinib | 2017/3/30 | EGFR | EGFR (T790M mutation) | Randomized | Targeted | no | Active treatment | yes |

Supplementary Table 1. List and the main characteristics of included FDA Drug Approvals (cont’d)

| No. | Drug | FDA approval date | Target molecule | Biomarker as eligibility criteria | Allocation | Class of agent | Concomitant medications | Control drug | Cross over |
| --- | --- | --- | --- | --- | --- | --- | --- | --- | --- |
| 21 | paclitaxel protein-bound particles | 2012/10/11 | Microtubules | No | Randomized | Cytotoxic | yes | Active treatment | no |
| 22 | pembrolizumab | 2016/10/24 | PD-1 | expressing PD-L1 [≥1%] | Randomized | Targeted | no | Active treatment | no |
| 23 | pembrolizumab | 2017/5/10 | PD-1 | No | Randomized | Targeted | yes | Active treatment | yes |
| 24 | pemetrexed disodium | 2004/8/19 | Folate-dependent metabolic processes | No | Randomized | Cytotoxic | no | Active treatment | no |
| 25 | ramucirumab | 2014/12/12 | VEGFR-2 | No | Randomized | Targeted | yes | Active treatment | yes |
| 26 | ramucirumab | 2020/5/29 | VEGFR-2 | EGFR (exon19 deletions or exon21(L858R) mutations) | Randomized | Targeted | yes | Active treatment | no |
| 27 | trametinib | 2017/6/22 | MEK1/2 | BRAF V600E mutation | non-randomized | Targeted | yes | Active treatment | no |

Supplementary Table 1. List and the main characteristics of included FDA Drug Approvals (cont’d)

| No. | Drug | Therapeutic line | First-in-class target in NSCLC drugs | Priority review | Orphan Drug Designations | Efficacy Parameter | Primary endpoint | Patient number |
| --- | --- | --- | --- | --- | --- | --- | --- | --- |
| 1 | afatinib | 1st line | no | PRIORITY | Orphan | OS, PFS  ORR, DoR | PFS | N=230 (afatinib)  N=115 (pemetrexed/cisplatin) |
| 2 | afatinib | Not 1st line | no | N/A | Orphan | OS, PFS  ORR | PFS | OS PFS, ORR  N=398 (afatinib) N=335 (afatinib)  N=397 (erlotinib) N=334 (erlotinib) |
| 3 | alectinib | 1st line | no | PRIORITY | Orphan | PFS, ORR  DoR | PFS | N=152 (alectinib)  N=151 (crizotinib) |
| 4 | atezolizumab | Not 1st line | yes | PRIORITY | N/A | OS, ORR  DoR | OS | N=144 (atezolizumab)  N=143 (docetaxel) |
| 5 | atezolizumab | 1st line | no | PRIORITY | N/A | OS, ORR  DoR | OS | N=359 (atezolizumab with bevacizumab, paclitaxel, and carboplatin)  N=337 (bevacizumab, paclitaxel and carboplatin) |
| 6 | bevacizumab | 1st line | yes | N/A | N/A | OS | OS | N=434 (bevacizumab, paclitaxel, carboplatin)  N=444 (paclitaxel, carboplatin) |
| 7 | cemiplimab | 1st line | no | PRIORITY | N/A | OS, PFS  ORR, DoR | OS, PFS | N=356 (cemiplimab)  N=354 (chemotherapy) |
| 8 | ceritinib | 1st line | no | PRIORITY | Orphan | PFS, ORR  DoR | PFS | N=189 (ceritinib)  N=187 (chemothrapy) |
| 9 | crizotinib | 1st line | yes | N/A | Orphan | OS, PFS  ORR, DoR | PFS | N=173 (crizotinib)  N=174 (chemothrapy) |

Supplementary Table 1. List and the main characteristics of included FDA Drug Approvals (cont’d)

| No. | Drug | Therapeutic line | First-in-class target in NSCLC drugs | Priority review | Orphan Drug Designations | Efficacy Parameter | Primary endpoint | Patient number |
| --- | --- | --- | --- | --- | --- | --- | --- | --- |
| 10 | dabrafenib | 1st line | yes | PRIORITY | Orphan | ORR, DoR | ORR | N=36 (dabrafenib, trametinib)  N=78 (dabrafenib only) |
| 11 | dacomitinib | 1st line | no | PRIORITY | Orphan | PFS, ORR  DoR | PFS | N=227 (dacomitinib)  N=225 (gefitinib) |
| 12 | durvalumab | Not 1st line | no | PRIORITY | N/A | PFS, ORR | OS, PFS | N=476 (durvalumab)  N=237 (placebo) |
| 13 | erlotinib | Not 1st line | yes | PRIORITY | N/A | OS, PFS  ORR, DoR | OS | N=488 (erlotinib)  N=243 (placebo) |
| 14 | erlotinib | 1st line | no | N/A | N/A | OS, PFS  ORR | PFS | N=86 (erlotinib)  N=88 (chemotherapy) |
| 15 | gefitinib | 1st line | no | N/A | Orphan | PFS, ORR  DoR | PFS, ORR | N=88 (gefitinib)  N=98 (carboplatin/paclitaxel) |
| 16 | ipilimumab | 1st line | yes | PRIORITY | N/A | OS, PFS  ORR, DoR | OS | N=396 (nivolumab, ipilimumab)  N=397 (platinum-doublet chemotherapy) |
| 17 | necitumumab | 1st line | no | N/A | Orphan | OS, PFS  ORR | OS | N=545 (necitumumab, gemcitabine, cisplatin)  N=548 (gemcitabine, cisplatin) |
| 18 | nivolumab | Not 1st line | yes | PRIORITY | N/A | OS | OS | N=135 (nivolumab)  N=137 (docetaxel) |
| 19 | nivolumab | 1st line | no | PRIORITY | N/A | OS, PFS  ORR, DoR | OS | N=396 (nivolumab, ipilimumab)  N=397 (platinum-doublet chemotherapy) |

Supplementary Table 1. List and the main characteristics of included FDA Drug Approvals (cont’d)

| No. | Drug | Therapeutic line | First-in-class target in NSCLC drugs | Priority review | Orphan Drug Designations | Efficacy Parameter | Primary endpoint | Patient number |
| --- | --- | --- | --- | --- | --- | --- | --- | --- |
| 20 | osimertinib | Not 1st line | no | PRIORITY | Orphan | PFS, ORR  DoR | PFS | N=279 (osimertinib)  N=140 (chemotherapy) |
| 21 | paclitaxel protein-bound particles | 1st line | yes | N/A | N/A | ORR, DoR | ORR | N=521 (paclitaxel protein-bound particles, carboplatin)  N=531 (paclitaxel, carboplatin) |
| 22 | pembrolizumab | Not 1st line | no | PRIORITY | N/A | OS, PFS  ORR, DoR | OS, PFS | N=344 (pembrolizumab)  N=343 (docetaxel) |
| 23 | pembrolizumab | 1st line | no | PRIORITY | N/A | PFS, ORR  DoR | ORR | N=60 (pembrolizumab, pemetrexed, carboplatin)  N=63 (pemetrexed, carboplatin) |
| 24 | pemetrexed disodium | Not 1st line | yes | N/A | N/A | OS, PFS  ORR | OS | N=283 (pemetrexed disodium)  N=288 (docetaxel) |
| 25 | ramucirumab | Not 1st line | yes | N/A | N/A | OS, PFS  ORR | OS | N=628 (ramucirumab, docetaxel)  N=625 (placebo, docetaxel) |
| 26 | ramucirumab | 1st line | no | N/A | N/A | OS, PFS  ORR | OS | N=224 (ramucirumab, erlotinib)  N=225 (placebo, erlotinib) |
| 27 | trametinib | 1st line | yes | PRIORITY | Orphan | ORR, DoR | ORR | N=36 (dabrafenib, trametinib)  N=78 (dabrafenib only) |

Supplementary Table 1. List and the main characteristics of included FDA Drug Approvals (cont’d)

| Drug | FDA approval date | Biomarker as eligibility criteria | Effect size | | |
| --- | --- | --- | --- | --- | --- |
|  |  |  | RRR | PFS HR | OS HR |
| afatinib | 2013/7/12 | EGFR mutations | 2.64 | 0.58 | 0.91 |
|  | 2016/4/15 | no | 1.50 | 0.82 | 0.81 |
| atezolizumab | 2016/10/18 | no | 1.00 | NA | 0.69 |
|  | 2018/12/6 | no EGFR or ALK mutations | 1.30 | 0.71 | 0.78 |
| erlotinib | 2004/11/18 | no | 9.89 | 0.59 | 0.73 |
|  | 2013/5/14 | EGFR mutations | 4.06 | 0.34 | 0.93 |
| nivolumab | 2015/3/4 | no | NA | NA | 0.59 |
|  | 2020/5/15 | PD-L1 expression, no EGFR or ALK mutations | NA | NA | 0.79 |
| pembrolizumab | 2016/10/24 | PD-L1 expression | 2.00 | 0.88 | 0.71 |
|  | 2017/5/10 | no | 1.90 | 0.53 | NA |
| ramucirumab | 2014/12/12 | No | 1.64 | 0.76 | 0.86 |
|  | 2020/5/29 | EGFR mutations | 1.01 | 0.59 | 0.83 |

Supplementary Table 2. List of effect sizes for drugs with both personalized and nonpersonalized indications

| The personalized strategy is used prior to NSCLC indication in the US | Indication is squamous NSCLC only | Orphan drug designation | Immuno-oncology target | First in class for the target | First line | Personalized | Drug |
| --- | --- | --- | --- | --- | --- | --- | --- |
| No | No | No | No | No | Yes | Yes | erlotinib |
| No | No | No | No | Yes | No | No | erlotinib, pemetrexed disodium, ramucirumab |
| No | No | No | No | Yes | Yes | No | bevacizumab, paclitaxel protein-bound particles |
| No | No | No | Yes | No | No | Yes | pembrolizumab |
| No | No | No | Yes | No | No | No | durvalumab |
| No | No | No | Yes | No | Yes | Yes | atezolizumab, cemiplimab, nivolumab |
| No | No | No | Yes | Yes | No | No | atezolizumab |
| No | No | No | Yes | Yes | Yes | Yes | ipilimumab |
| No | No | Yes | No | No | No | Yes | osimertinib |
| No | No | Yes | No | No | Yes | Yes | afatinib, ceritinib, dacomitinib |
| No | No | Yes | No | Yes | Yes | Yes | crizotinib |
| No | Yes | No | Yes | Yes | No | No | nivolumab |
| No | Yes | Yes | No | No | Yes | No | necitumumab |
| Yes | No | No | No | No | Yes | Yes | ramucirumab |
| Yes | No | No | Yes | No | Yes | No | pembrolizumab |
| Yes | No | Yes | No | No | Yes | Yes | alectinib, gefitinib |
| Yes | No | Yes | No | Yes | Yes | Yes | dabrafenib, trametinib |
| Yes | Yes | Yes | No | No | No | No | afatinib |

Supplementary Table 3. Truth table analysis for QCA
